# Peripheral Sensory Neuropathy and associated factors among adult diabetes mellitus patients in Bahr Dar, Ethiopia

**DOI:** 10.1186/s40200-017-0295-5

**Published:** 2017-04-04

**Authors:** Gashaw Jember, Yayehirad Alemu Melsew, Berihu Fisseha, Kedir Sany, Asmare Yitayeh Gelaw, Balamurugan Janakiraman

**Affiliations:** 1 Department of physiotherapy, School of Medicine, College of Medicine and Health Sciences, University of Gondar, Gondar, Ethiopia; 2 Department of Epidemiology and Biostatistics, Institute of Public Health, College of Medicine and Health Sciences, University of Gondar, Gondar, Ethiopia; 3 Department of Physiotherapy, School of Medicine, College of Health Sciences and Ayder comprehensive specialized Hospital, Mekelle University, Mekelle, Ethiopia

**Keywords:** Diabetes peripheral sensory neuropathy (DPSN), Diabetes mellitus, Ethiopia

## Abstract

**Background:**

Diabetic sensory neuropathy is a common form of microvascular complication among diabetic patients. The swiftly growing population of people living with diabetes in Ethiopia and lack of elaborated scientific data on peripheral sensory neuropathy among diabetic population in Ethiopia prompted this work. This study was set out to assess the enormity and associated factors of peripheral sensory neuropathy among diabetes patients attending chronic illness clinic of Felege Hiwot Regional Referral Hospital, Bahr Dar, Northwest Ethiopia.

**Methods:**

An institution based cross-sectional study was conducted at Felege Hiwot Referral Hospital chronic illness clinic using Michigan neuropathy screening instrument tool for diabetic peripheral sensory neuropathy on 408 diabetic patients during 2016. Data were collected using interview, patient record review, anthropometric measurements and physical examination. Both bivariate and multivariate binary logistic regression was employed to identify factors associated with peripheral sensory neuropathy. Odds ratios with their 95% CI and *P* value less than 0.05 used to determine statistically significant associations.

**Results:**

A total of 368 patients were included with the mean age of 49 ± 14.3 years. The overall prevalence of Peripheral Sensory Neuropathy was found to be 52.2%. The major associated factors identified by multivariate analysis were age >50 years: AOR: 3.0 CI [1.11, 7.89]; overweight and obese: AOR: 7.3 CI [3.57, 14.99]; duration of DM: AOR: 3.4 CI [1.75, 6.60]; not involved in physical exercise: AOR: 4.8 CI [1.90, 7.89]; male gender: AOR: 2.4 CI [1.18, 5.05].

**Conclusion:**

Almost half of the diabetic patients who attended Felege Hiwot regional referral hospital during study period were found to present with peripheral sensory neuropathy. Socio-demographic and bio characteristics like patients age, Body Mass Index, level of physical activity and marital status were significantly associated with diabetic peripheral sensory neuropathy.

## Background

Diabetes mellitus (DM) is swiftly emerging health problem in developing countries; like so in Ethiopia. Due to its chronic nature, it causes many debilitating complications including neuropathy, retinopathy, and nephropathy and macro vascular disease [1].

**Fig. 1:**
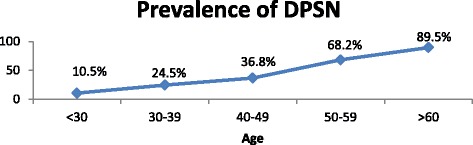
Trend of DPSN to increase in age among adult diabetic participants at Bahr Dar Felege Hiwot Referral Hospital, 2016, (*n* = 368)

Peripheral sensory neuropathy (PSN) is one of the complications of diabetes and the most common cause of foot ulceration and amputations. It involves damage to the nerve fibers or entire nerve cells. Some diabetic peripheral neuropathic patients may have extremely painful symptoms, others may be asymptomatic [2]. So the absence of symptoms may not be equated with absence of neuropathy which leads to risk of developing foot ulcers and amputations resulting huge impact on reducing the health related quality of life of DM patients [3–5].

The global estimates of Diabetic peripheral sensory neuropathy (DPSN) prevalence vary widely from 9.6 to 88.7% in different populations. This may be attributed to different types of diabetes, existing health care facilities, sample selection, different diagnostic criteria used, variable methods etc. [6–8].

Patients with DPSN might suffer from pain and discomfort in the lower extremities, loss or absence of protective sensation in the lower extremities leading to balance problems, risk of foot ulcerations, and a reduced quality of life in adults with diabetes patients [9].

Most of the time nerve damage and DPSN are irreversible. Hence, early screening of DPSN and identifying associated factors is very crucial especially for developing countries with low resource settings and low literacy rate [5].

The most common factors that may influence the DPSN are age, duration of diabetes since diagnosis, poor glycemic control; history of ulcer; education level, smoking habit, drinking alcohol and body mass index directly increases the incidence of DPSN [7, 10, 11].

However, in North West Ethiopia the prevalence and associated factors of peripheral sensory neuropathy has not yet been elaborated among Northwest Ethiopian diabetic population.

Therefore this study is aimed to assess the prevalence and associated factors of diabetic peripheral sensory neuropathy among adults with diabetes mellitus in Felege Hiwot Regional Referral Hospital, Northwest Ethiopia.

## Method

### Study setting

An Institution based cross-sectional study was conducted from February 2016 to June 30 2016. The study was done at the diabetes unit of chronic illness clinic of Felege Hiwot Referral Hospital, North West Ethiopia.

### Study participants

The study population comprised of diabetic patients who were under routine medical supervision/follow up and attending out-patient diabetic unit of chronic illness clinic of Felege Hiwot Referral Hospital. Both type I and II diabetes mellitus; age ≥ 20 years were included and those who were seriously ill, DM patients with HIV, leprosy, peripheral nerve injury, bilateral lower extremity amputees were excluded based on medical chart, history and baseline test.

### Sample size determination

The sample size was determined using EPI INFO seven program to estimate a single population proportion based on the following assumptions: 95% level of confidence interval and 5% margin of error. The proportion of DPSN among diabetic patients was taken as 41% from a study done in Mekele, Ethiopia [12]. With 41% assumption the derived sample size was 371 and an addition of 10% non response rate made it (n) 408.

### Sampling procedure

Systematic random sampling technique was used to recruit participants. The pattern of patient flow rate in the diabetic clinic was considered. As a result, the average patient flow rate was known to be 180 per week. The average flow rate was matched with six weeks of data collection period and a total of 1080 diabetic patients were assumed to be available during this period. The sample was taken from relatively small population (*N* = 1080). The patients included in the sample were selected every third interval. The first patient was selected randomly from the first three by a lottery method, and the next patient was interviewed every third interval.

### Variables of the study

The dependent variable was diabetic peripheral sensory neuropathy measured by Michigan neuropathy screening instrument (MNSI) tool. The independent variables included in the study were; Socio-demographic characteristics, behavioral characteristics and clinical characteristics. The intervening and confounding variables which could influence DPSN are oral anti diabetic agents, insulin, glycemic control (HbA_1_C), foot care practice and cardiovascular comorbidities.

### Operational definition

The diabetic patient has been considered as having peripheral sensory neuropathy if the patients history version of MNSI questionnaire scoreis ≥7 abnormal responses in the legs (feet) and/or if the lower extremity examination version of MNSI scores ≥2.5 in the legs (feet).

### Data collection instrument

A structured questionnaire was prepared for interview to collect the socio-demographic information. Clinical characteristics were collected from the patient records. Patients were diagnosed for sensory neuropathy by using the MNSI tool. This tool consists of two parts, the first part is history version that contains a questionnaire administered by the interviewer and the second part contains physical assessment examination performed by physiotherapists. The physical examination was done by using multiple 10 gm MEDLINE monofilaments for diabetic neuropathy foot test made in USA to detect diminished or loss of sensation. Each monofilaments were used to test a maximum of 10 patients to avoid over diagnosing error resulting from loss of viscoelasticity due to frequent buckling.

A 128 HZ American Diagnostic Corporation (ADC) Tuning fork was used to detect diminished or loss of vibration sense and Reflex hammers were used to elicit ankle reflex/Achilles tendon reflex to detect the quality of stretch reflex pathway function. The English version of Michigan neuropathy screening instrument was translated to the local language (Amharic) and again translated back to English by language experts. The original and translated questionnaires were compared and the discrepancies were reviewed and resolved accordingly.

### Data collection methods

Data were collected through face-to-face interviews, patient record interviews and by physical examination. Anthropometric measurements were conducted to measure height and weight of the study subjects. BMI was calculated as kg/m^2^ to determine the nutritional status of body composition. Data collection was carried out by a nurse and two other physiotherapists. A 3 days intensive training was given to the data collectors by the principal investigator (GJ) on how to approach the study participants, how to use the questionnaire and guideline, and the techniques of data collection. The investigators closely followed the data collection process and ensured accuracy, completeness and consistency of the collected questionnaire daily.

### Data quality control Methods

To ensure the quality of data and cultural competence of the tool, pretests of data collection tools were carried out on 45 diabetes patients attending Gondar university Hospital Diabetic Clinic prior to actual data collection and those subjects were excluded from the study. After analyzing the pretest results, necessary modifications and corrections were made accordingly before using the tools in the actual survey. Every day the collected questionnaire was checked for clarity, consistency, and completeness by the investigators. Consequently, amendments and corrections were made before the start of the next day’s work. Data double entry was done for reliability and correctness with the respective original data. In addition, computer data cleaning was done.

### Data analysis

Data were coded and entered using EPI INFO version 7 and exported to SPSS for windows version 20. Missing values and data cleaning were checked. Descriptive statistics for the presentation of demographic data, including percentage, or mean and standard deviation (SD) was used. Binary logistic regression was used to identify predictors of DPSN among diabetes patients. Variables with a bivariate *P* < 0.20 were fitted into multivariate models for controlling the possible effect of confounders, and finally the variables that had significant association with DPSN were identified on the basis of odds ratio (OR), with 95% confidence interval (CI) and *P* < 0.05. The variables were entered into the multivariate model using the backward stepwise (likelihood ratio) method.

## Result

### Socio-demographic characteristics

A total of 368 diabetic patients participated in the study with a response rate of 90.2%. Forty adult diabetic patients who were selected as sample could not participate in the study due to unwillingness, incomplete medical chart and refusing to take baseline test. The mean age of the participants was 49 years (±14.3). Majority of the respondents were males (58.4%), were married (80.2%) and urban dwelling (82.9%). Many participants were orthodox Christians (61.7%) and almost One third (33.4%) of them were governmental employee. The socio-demographic details of the participants have been provided in Table 1.

**Table 1:** Sociodemographic characteristics and prevalence of DPSN of participants at Bahr Dar Felege Hiwot regional referral Hospital, April 2016, (*n* = 368)

Variable		Frequency (%)	Outcome of DPSN	
			Yes (%)	No (%)
Sex	Female	153 (41.6)	87 (56.9)	66 (43.1)
	Male	215 (58.4)	104 (48.4)	111 (51.6)
Residence	Urban	305 (82.9)	167 (54.8)	138 (42.2)
	Rural	63 (17.1)	24 (38.1)	39 (61.9)
Religion	Orthodox	227 (61.7)	104 (45.8)	123 (54.2)
	Muslim	115 (31.3)	74 (64.3)	41 (35.7)
	Protestant	24 (6.5)	12 (50)	12 (50)
	Catholic	2 (0.5)	2 (100)	0.0
Marital status	Married	295 (80.2)	158 (53.6)	137 (46.4)
	Unmarried	73 (19.8)	34 (46.6)	39 (53.4)
Educational status	No formal.	22 (6.0)	15 (68.2)	7 (31.8)
	Elementary [1–8]	44 (12)	24 (54.5)	20 (45.5)
	High school [9–12]	142 (38.6)	85 (59.9)	57 (40.1)
	College and above	160 (43.5)	68 (42.5)	92 (57.5)
Occupational status	Govt. employ	123 (33.4)	43 (35.0)	80 (38.0)
	Private employ	107 (29.1)	66 (61.7)	41 (38.3)
	Housewife	35 (9.2)	24 (70.6)	10 (29.4)
	Unemployed	12 (3.3)	3 (25.0)	9 (75.0)
	Farmer	39 (10.6)	15 (38.5)	24 (61.5)
	Others	53 (14.4)	40 (75.5)	13 (24.5)
Income	<1000	124 (33.7)	68 (54.8)	56 (45.2)
	1000–1999	27 (7.3)	21 (77.8)	6 (22.2)
	2000–2999	39 (10.6)	20 (51.3)	19 (48.7)
	≥3000	178 (48.4)	82 (46.1)	96 (53.9)
Alcohol consumption	Yes	169 (45.9)	96 (56.8)	73 (43.2)
	No	199 (54.1)	95 (47.7)	104 (52.3)
Smoking status	Yes	26 (7.1)	26 (100)	0
	No	342 (92.9)	165 (48.2)	177 (51.8)
Type of DM	Type 1	164 (44.6)	84 (51.2)	80 (48.8)
	Type 2	204 (55.4)	107 (52.5)	97 (47.5)
Duration of DM	≤10 yrs	190 (51.6)	56 (29.5	134 (70.5)
	≥10 yrs	178 (48.4)	135 (75.8)	43 (24.2)
Hypertension	Yes	129 (35.1)	103 (79.8)	26 (20.2)
	No	239 (64.9)	88 (36.8)	151 (63.2)

### The relation between Age of study participants and peripheral senseory neuropathy

An increasing trend in prevalence of PSN with increasing age was observed (Fig. 1).

### Clinical and Behavioral characteristics

A total of 263 (71.5%) of study participants were in normal weight category on BMI whereas 105 (28.6%) participants were overweight or obese. Most (55.4%) of the participants were type 2 diabetes mellitus. One hundred ninety (51.6%) of the study participants’ were known to be diagnosed with diabetes for less than 10 years and majority (64.9%) of the participants had no history of hypertension. Most of the participants (92.9%) were non-smokers and 45.9% of them reported to have habit of alcohol consumption. The remaining clinical and behavioral details of the participants are presented in Table 2.

**Table 2:** Clinical and behavioral characteristics of patients with diabetes mellitus at Bahr Dar Felege Hiwot referral regional Hospital, 2016, Bahr Dar, Ethiopia (*n* = 368)

Variables		Frequency (%)
BMI	<25	263 (71.5)
	25–29	79 (21.5)
	≥30	26 (7.1)
Types of DM	Type 1	164 (44.6)
	Type 2	204 (55.4)
Duration of DM	<10	190 (51.6)
	≥10	178 (48.4)
Hypertension	yes	129 (35.1)
	no	239 (64.9)
Involved in physical exercise	yes	98 (26.6)
	no	270 (73.4)
Alcohol consumption	yes	169 (45.9)
	no	199 (54.1)
Smoking status	yes	26 (7.1)
	no	342 (92.9)

### Prevalence of DPSN among diabetic patients

The overall prevalence of diabetic peripheral sensory neuropathy was found to be 192 (52.2%) among the study population. Among this, the prevalence of DPSN among type1 diabetes patients was found to be 51.2%. The prevalence of DPSN was slightly higher among diabetic women (56.9%) than men (48.4%). The prevalence of DPSN based on other socio demographic characteristics are expressed in Table 1.

### Factors associated with DPSN

Variables that were significantly associated with DPSN in bivariate analysis were reentered into multivariate logistic regression model as independent variables for outcome of DPSN. The factors that were identified to be significantly associated with the development of DPSN were; increased age, being overweight or obese, longer duration of DM and lack of physical activity.

Diabetic patients who were 50 and above years old are about four times more likely to have DPSN than younger patients [AOR = 4.17, 95% CI: 1.60. 10.85]. Diabetic patients who were not involved in regular physical activities were eight times more likely to develop DPSN than those who had regular physical exercise program [AOR: 8.2, CI [3.40, 19.56]. Being overweight or obese was found to be alarmingly [AOR: 16.3, CI [6.12, 43.43] associated with PSN. With duration of diabetes; patients living with DM for 10 years and more were found to be at risk of developing PSN about four times AOR: 4.5, CI [2.07, 9.64] more than those with duration of DM less than 10 years (Table 3).

**Table 3:** factors associated with DPSN among diabetic patients at Bahr Dar Felege Hiwot regional referral Hospital by bivariate and multivariate logistic regression analysis, April 2016, Bahr Dar Ethiopia (*n* = 368)

Variable		DPSN (n)		Crude OR (95% CI)		AOR (95% CI)	
		Yes	No				
Age	<40	20	86	1		1	
	40–50	44	60	3.2	1.69, 5.88	4.2^a^	1.60, 10.85
	>51	127	31	17.62	9.43, 32.92	66.1^a^	18.59, 235.32
Sex	Female	87	66	1		1	
	Male	104	111	0.7	0.47, 1.08	2.1	0.97, 4.62
Educational status	No formal	15	7	0		0	
	Grade 1–8	24	20	2.776	1.063, 7.251	2.122	0.571, 7.886
	Grade 9–12	85	57	1.666	0.851, 3.260	1.786	0.740, 4.312
	College & a	68	92	1		1	
Income	<1000	68	56	1.422	0.897, 2.253	1.861	0.643, 5.384
	1000–1999	21	6	4.098	1.578, 10.637	3.986	0.822, 19.328
	2000–2999	20	19	1.232	0.616, 2.466	1.628	0.456, 5.806
	≥3000	82	96	1		1	
Physical exercise	yes	71	26	1		1	
	no	106	165	4.3	2.57, 7.16	8.2^a^	3.40, 19.56
Alcohol consumption	yes	96	73	1.440	0.953, 2.174	1.155	0.555, 2.406
	no	95	104	1		1	
Duration of DM	<10 yrs	56	134	1		1	
	≥10 yrs	135	43	7.5	4.73, 11.95	4.5^a^	2.07, 9.64
Hypertension	yes	103	26	6.798	4.106, 11.254	0.898	0.289, 2.793
	no	88	151	1		1	
BMI	<25	100	163	1		1	
	25–29	69	10	11.3	5.54, 22.84	16.3^a^	6.12, 43.43
	≥30	22	4	9.0	3.00, 26.77	28.4^a^	6.47, 124.28

^a^Significantly associated with *p* < 0.05

## Discussion

This study found out that the prevalence of DPSN according to the MNSI patient history version was 36.3%, which is much lesser than prevalence explored by MNSI examination which was 52.2%. The above difference clearly demonstrates the limitations related to patients self perception of symptoms of DPSN and suggest insisting on MNSI examination to be administered regularly during out-patient visit. Similar finding was observed in a study conducted in Logos Nigeria [13]. The prevalence of DPSN using MNSI examination among adult diabetic participants in.

Bahr Dar Felege Hiwot Regional Referral Hospital was found to be 52.2% which was more similar to the findings of other studies conducted in Iran and University of Washington, USA [8, 14] which reported a prevalence of 53.3 and 50% respectively. The similarity could be due to the reason that all studies used MSNI examination tool. The prevalence found in this study falls within the range reported by systematic reviews [12, 15, 16]. However, the range reported by those studies was much wider ranging from 26% to 77%.

The difference in the prevalence of DPSN across the studies maybe attributed to differences in study design, type of population included, resource of hospital set up and type of tool used to assess the magnitude of DPSN in different study settings.

Among studies done in Ethiopia this study reported a higher prevalence of 52.2% when compared with 41 and 25% by studies done in Mekele and Jimma respectively [17, 18]. The proposed reason could be; the Mekele study was a prospective cohort study detecting only new cases where as this study is a cross sectional one where new and old cases were detected. The Jima study used only a structured questionnaire but this study MSNI history version and examination version which helped identifying more cases. Studies in United Arab Emirates, India, South Africa and Nigeria reported a prevalence of 30.3%, 10.5, 37, 32.2% respectively [3, 6, 7, 13] which are lower than the prevalence of DPSN in the present study. The possible explanation for the difference in prevalence of DPSN might be the difference in study population, tools used and methodologies. For example, South African study used Douleur neuropathic questionnaire (DN_4_) tool which was different from MNSI tool used in this study and the Nigerian study included diabetes patients diagnosed before five years unlike to the present study which included all the diabetic patients attending at the time of data collection.

Our findings were also lower than the study done in USA (66.7%), Benin (88.7%) and Taiwan (71%) [19–21]. The differences in prevalence could possibly be due to differences in outcome tool and patient characteristics. For instance, the Seattle, USA study used Nerve Conduction Velocity test (NCV) which is a golden standard, costly, time consuming and found in high resource set ups whereas the present study used MNSI tool which is easily applicable and cheap. The study done in Benin used diabetic neuropathy score (DNS) tool and investigated only patients with neuropathic symptoms unlike the present study which investigated all symptomatic and asymptomatic diabetic patients with MNSI. Others studied patients with neurological signs [1, 22] and some studies also combined both neuropathic symptoms and neurological signs similar with this study [15, 17, 21]. Some studies included neurological complications of diabetes patients, motor, sensory and autonomic nerves whereas other authors combined both patients with foot ulcers and peripheral neuropathy [14, 17, 18]. Therefore, the disparities in reported prevalence would have occurred due to factorial differences like type of DM, age group, outcome tools,sample size and socio demographic characteristics of patients.

After conducting multivariate logistic regression, we observed that age above 50 years were found to be significantly associated with the development of DPSN. Which is imilar to findings reported in studies conducted in University of Alfenas, Brazil, Fasa University, Hamedan province, Iran, Ethiopia, Benin, USA, Nigeria respectively [5, 10, 14, 15, 21, 23, 24]. Aging is a well known risk factor for many non-communicable diseases including DM and increasing age is a risk factors for many neurological disorder, probably reflecting the limited regenerative capacity of the nervous system after any insult. The effect of aging combined with deleterious effects of chronic hyperglycemia can result in increased prevalence of DPSN as one gets older. Other possible reasons could be; peripheral nerve system by their length and size are known to have increased vulnerability with aging due to continual metabolic stress and degenerative nature of physiological well-being.

In the present study, for those who did not involve in physical exercise (sedentary life style) was found to be significantly associated with the development of DPSN in multivariate analysis. Similar findings were observed in studies conducted in Brazil and India [25, 26], However no such association was observed in other studies conducted in India, Iran, Benin, Taiwan [10, 14, 20, 21] The reason behind might be physical exercise can increase micro-vascular circulation, prevent aging, boosts immune system, gives strength to muscle and cardiovascular system as well as physiological well-being of the body so as to negatively affect the occurrence of DPSN.

Increased body mass index was significantly associated with the development of DPSN in diabetes patients in multivariate analysis. This is comparable to the results of studies in South Africa, Ethiopia, Logos, Nigeria, Taiwan USA, UAE [6, 7, 18–20, 26]. The authors also reported that co morbid like hypertension, peripheral vascular diseases and increasing BMI by itself can proportionally increase the risk of DPSN among DM patients.

Duration of diabetes mellitus for more than 10 years were found to be significantly associated with the development of DPSN in diabetic patients in multivariate analysis. Similar to the findings obtained in Iran, India and Brazil [19, 20, 26] respectively. Longer duration or chronic hyper glycaemia might result in glycosylation of nervous tissue and therefore damage. Smoking was not significantly associated with DPSN in this study. Only 7.1% of the participants reported as smokers. But all of them were found to have DPSN. This might be due to small number of smokers in the study population. Unlike the present study, most of the studies done [9, 25, 27] on the developments of DPSN reported association with the cigarette smoking.

Unlike the present study, low education level, low income, ethnicity being black, presence of hypertension, and male gender were significantly associated with the incidence of DPSN in studies done in Iran, Benin, Bangladesh, Taiwan, and Ethiopia respectively [12, 18, 20, 21, 26]. The possible reason behind this might be the methodology of the study, study area, and difference in sample size of the study.

### Limitation

This study certainly has few limitations like sample size being not enormous; which was evident by wider confidence interval. Golden standard diagnostic procedure like Nerve Conduction Velocity Test (NCV) was not performed due to non-affordability.

The effects of intervening and confounding variables like oral anti diabetic agents, insulin, glycemic control (HbA_1_C), foot care practice and cardiovascular comorbidities on DPSN were not studied.

## Conclusions

This study shows high prevalence of DPSN that almost half of diabetes mellitus patients had experiences DPSN.

Among diabetes patients older age (above >50 years); body mass index (>25 kg/m^2^); male gender; longer duration of DM and not doing physical exercise were found to be significant factors associated with development of DPSN.

It is worthwhile if Physiotherapists and other health care professionals have an integrated, multidisciplinary and need based approach to prevent DPSN and if comprehensive examination of sensory neuropathy is included as an integral part of DM care. It is also vital that patients living with diabetes mellitus follow regular physical exercise in order to reduce the incidence of DPSN.

## Abbreviations

BMI: Body mass index
DM: Diabetics mellitus
DPSN: Diabetic peripheral sensory neuropathy
MNSI: Michigan neuropathy screening instrument
NCVT: Nerve Conduction Velocity Test
PSN: Peripheral sensory neuropathy
